# The ART of Resistance

**DOI:** 10.1080/27694127.2022.2134254

**Published:** 2022-10-26

**Authors:** Ananya Ray, Namita Surolia

**Affiliations:** Molecular Biology and Genetics Unit, Jawaharlal Nehru Centre for Advanced Scientific Research, Bengaluru, 560064, India

**Keywords:** Artemisinin resistance, autophagy, ER stress, Kelch13, *P. falciparum*, *Pf*ATG18, unfolded protein response

## Abstract

Recent emergence and spread of artemisinin (ART) resistance in South-east Asia caused by mutations in *P. falciparum* Kelch13 in the background of other mutations including mutations in the macroautophagy/autophagy-related protein *Pf*ATG18, has intensified studies towards understanding the molecular mechanisms of resistance. The autophagy pathway of the parasite has been hypothesized to engage in resistance-associated proteostasis involving enhanced phosphatidylinositol-3-phosphate vesiculation, oxidative stress, unfolded protein response and also reduced hemoglobin endocytosis resulting from nutrient-limiting conditions, albeit without any experimental evidence. We demonstrate that ART-induced ER stress leads to upregulation of parasite autophagy through the unfolded protein response pathway. In addition, we show elevated basal expression of autophagy proteins in the ART resistant Kelch13^C58^°^Y^ isolate as compared to its isogenic counterpart WT Kelch13. When autophagy is induced through starvation, the expression levels of autophagy proteins increase further in the resistant parasites. The decreased IC50 of the autophagy-specific inhibitor MRT68921 in resistant parasites relative to its isogenic counterpart establishes that autophagy is the key parasite survival mechanism in ART resistance. Additionally, upon analyses of *Pf*Kelch13 mutations from various field isolates, we observe a clear association between *Pf*Kelch13 (C580Y, R539T and Y493H) and *Pf*ATG18 (T38I) mutations. The copresence of *Pf*ATG18 with *Pf*Kelch13 on parasite cytostome-like and hemoglobin-containing vesicles provides further evidence that autophagy underpins various mechanisms of ART resistance.

**Abbreviations:** ART, artemisinin; DHA, dihydroartemisinin; eIF2A, eukaryotic translation initiation factor subunit eIF2A; ER, endoplasmic reticulum; PtdIns3P, phosphatidylinositol-3-phosphate; *Pf*ATG18, *P. falciparum* autophagy-related protein 18; *Pf*ATG8, *P. falciparum* autophagy-related protein 8; *Pf*SEC62, *P. falciparum* translocation protein SEC62; PK4, *Plasmodium* eIF2A kinase; WT, wild type.

Artemisinin-based combination therapies have significantly reduced the malaria burden and deaths globally. These first-line treatments combine a long-acting partner drug (pyronari-dine, sulfadoxine-pyrimethamine, mefloquine, lumefantrine, piperaquine) with a fast-acting and effective antimalarial derivative of artemisinin (ART) such as artesunate, artemether and dihydroartemisinin (DHA). Malaria parasites have, however, begun to develop resistance to ART, posing a threat to the eradication of the disease. The diminishing effectiveness of artemisinin-based combination therapies against *P. falciparum* raises concerns that ART resistance may evolve and spread to malaria-endemic regions. Current understanding of the mechanisms of *P. falciparum* by which parasites eventually develop resistance to ART is limited.

ART induces widespread cellular damage by alkylating proteins promiscuously, resulting in the buildup of misfolded proteins in the parasite ER and cytoplasm. This results in proteopathy leading to disruption of various parasite metabolic functions and viability. Protein homeostasis, involving proper protein folding and vesicular remodeling (proteostasis), as well as reduced hemoglobin endocytosis are the two major mechanisms proposed to be responsible for ART resistance. Several point mutations in *Pf*Kelch13 in association with mutations in other proteins such as the autophagy-related protein *Pf*ATG18 have been identified as the genetic markers for ART resistance, with the *Pf*Kelch13^C58^°^Y^ mutation being the most prevalent. It has been suggested that mutations in *Pf*Kelch13 mitigate ART-induced proteopathy through proteostatic dysregulation of phosphatidylinositol 3-kinase, leading to elevation of phosphatidylinositol-3-phosphate (PtdIns3P) and subsequent expansion of homeostatic ER-PtdIns3P vesicles of proteostasis. Amplified PtdIns3P vesicles disseminate proteostatic capacity throughout the parasite, which may prevent ART-induced parasite death. Reduced hemoglobin endocytosis during the early ring stage of the parasite’s asexual cycle also forms the basis for the *Pf*Kelch13-mediated ART resistance. Studies have provided evidence that *Pf*Kelch13 is also involved in hemoglobin endocytosis in the parasite. The major mutation in *Pf*Kelch13 impairs *Pf*Kelch13 levels, which significantly reduces hemoglobin endocytosis and degradation. The decreased availability of hemoglobin confers ART resistance as hemoglobin-derived iron is necessary to activate ART. Though each of these pathways, as well as PtdIns3P vesiculation, can independently induce autophagy, a direct demonstration for the involvement of autophagy in resistance is lacking.

Autophagy is a self-degradative process, engaged in various stress responses such as starvation, hypoxia and oxidative stress; providing macromolecules to recycle and mediating cell homeostasis. Autophagy proteins function in concert to maintain protein quality control by degrading misfolded proteins in the lysosome of eukaryotic cells. ER stress-mediated activation of unfolded protein response (UPR) signaling transducers regulates autophagy at various stages, including induction, phagophore nucleation, and phagophore expansion. Along with a robust ER stress response mechanism, the parasite genome also encodes a small number of partially conserved autophagy-related proteins that have been implicated in autophagy-like processes, it is, however, not known whether the parasite autophagy pathway would be activated in response to ER stress. Additionally, a specific mutation (T38I) in *Pf*ATG18 is strongly selected under ART resistance and confers a fitness advantage to parasites by allowing for faster growth rates under nutrient-limited conditions. Our findings show that 2517 *P. falciparum* genome isolates from Southeast Asia and Africa share the T38I mutation in *Pf*ATG18 and the C580Y mutation in *Pf*Kelch13 [1]. The wild-type *Pf*ATG18 is also thought to be phosphorylated at the T38 position. *Pf*ATG18 binds to and utilizes PtdIns3P for membrane association. We speculate that the mutation in *Pf*ATG18 could be selected during ART resistance to compensate for the parasite fitness loss associated with resistance by increasing its association with PtdIns3P, thereby promoting autophagy in the parasite. Involvement of parasite autophagy in proteostasis mechanisms of ART resistance to a large extent remains unreported.

In our recent work, we investigated the cross-talk between ER stress, UPR and the autophagy-like pathway in the malaria parasite. We found that DHA (the active ART metabolite)- mediated proteostatic stress increases eIF2A phosphorylation, reducing protein translation and promoting dormancy. Live cell imaging with *Pf*SEC62-GFP, an ER membrane-localized parasite protein, reveal that the parasite ER does expand following DHA exposure, an indication of ER stress. Induction of autophagy upon DHA treatment is demonstrated by an increase in the number of *Pf*ATG8-labeled autophagosome-like vesicles and the expression levels of two parasite autophagy-related proteins, *Pf*ATG8 and *Pf*ATG18. Inhibition of PK4-eIF2A-mediated UPR signaling decreases *Pf*ATG8 and *Pf*ATG18 expression levels, further validating our proposition that ER stress-induced autophagy is mediated through the UPR.

Further we provide novel insights into the role of parasite autophagy in ART resistance mechanisms. The transcript and the protein levels of *Pf*ATG8 and *Pf*ATG18 are higher in the ART-resistant Kelch13^C58^°^Y^ isolate as compared to its isogenic WT Kelch13 isolate. Also, the resistant parasites show increased colocalization of *Pf*ATG18-labeled vesicles with the autophagosome marker *Pf*ATG8 as compared to the isogenic one, indicating activated autophagy in the parasites even at the basal level. Amino acid starvation further activates autophagy in both WT Kelch13 and Kelch13^C58^°^Y^ isolates, although the colocalization of *Pf*ATG18 and PtdIns3P-labeled vesicles is increased in ART-resistant parasites compared to isogenic parasites. A dose-response analysis of the specific autophagy inhibitor MRT68921 indicates a 2-fold reduction in the inhibitor’s IC50 in ART-resistant parasites compared to its isogenic counterpart. Importantly, we find that *Pf*Kelch13, along with *Pf*ATG18, is transported to the food vacuole via hemoglobin-containing vesicles, which transport host-derived hemoglobin to the parasite food vacuole.

Altogether, we find a previously unexplored interplay between ART-mediated ER stress, UPR, and autophagy in *P. falciparum*, as well as the contribution of parasite autophagy to the establishment of ART resistance (Figure 1). Our findings thus highlight the potentials of inhibiting the parasite autophagy as a target to develop antimalarials for resistant *P. falciparum* infection.

**Figure 1.**
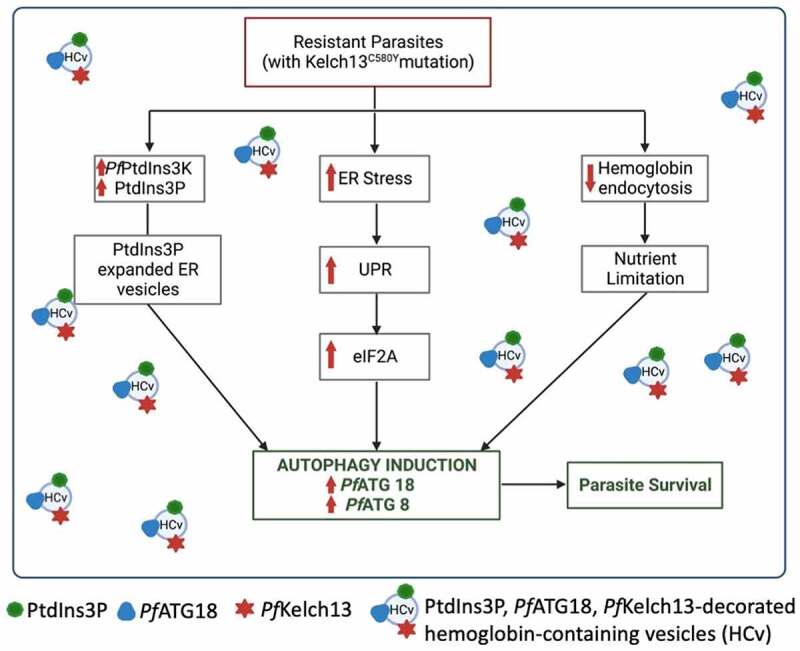
Autophagy mediates ART resistance in *P. falciparum*.
